# Modification of the association between coffee consumption and constipation by alcohol drinking: A cross-sectional analysis of NHANES 2007–2010

**DOI:** 10.1371/journal.pone.0311916

**Published:** 2024-10-25

**Authors:** Wanru Kong, Wei Sheng, Ya Zheng

**Affiliations:** 1 Department of Infection Management, Gansu Provincial Hospital, Lanzhou, Gansu, China; 2 School of Public Health, Xiamen University, Xiamen, Fujian, China; 3 Department of Gastroenterology, The First Hospital of Lanzhou University, Lanzhou, Gansu, China; 4 Gansu Province Clinical Research Center for Digestive Diseases, The First Hospital of Lanzhou University, Lanzhou, Gansu, China; University of Hawai’i at Manoa College of Tropical Agriculture and Human Resources, UNITED STATES OF AMERICA

## Abstract

**Background:**

The association between coffee consumption and constipation remains unclear. This study aimed to examine the relationship of coffee consumption with the risk of constipation, while also investigating potential effect modifiers.

**Methods:**

This cross-sectional study included 7844 participants from the National Health and Nutrition Examination Survey (NHANES) 2007–2010. Coffee consumption was extracted from the 24-hour dietary recall. Constipation was assessed using the Bristol Stool Form Scale. The association between coffee consumption and constipation was assessed using multivariable restricted cubic spline and logistic regression with odds ratio (OR) and 95% confidence interval (CI).

**Results:**

There was a J-shaped relationship between total coffee consumption and the risk of constipation in the whole population (p for nonlinearity = 0.049), with 1–2 cups/day of total coffee potentially reducing the risk of constipation by 39% (OR 0.61, 95% CI 0.35–1.06, p = 0.07). As for caffeinated coffee, a J-shaped association between its consumption and the risk of constipation was also observed in the whole population (p for nonlinearity = 0.008), with 1–2 cups/day being significantly associated with a reduced risk (OR 0.57, 95% CI 0.35–0.95, p = 0.03). When restricting to never drinkers of alcohol, the associations between total and caffeinated coffee consumption and constipation shifted to inverse linear trends, where at least 3 cups/day was significantly associated with an 88% reduction in constipation risk (total coffee: OR 0.12, 95% CI 0.02–0.68, p = 0.02; caffeinated coffee: OR 0.12, 95% CI 0.02–0.70, p = 0.02). Decaffeinated coffee showed no association with constipation.

**Conclusions:**

Consuming 1–2 cups of caffeinated coffee daily was associated with a reduced risk of constipation in the general population. Among never drinkers of alcohol, a linear protective effect was observed, with a notable 88% reduction in constipation risk for those consuming at least 3 cups per day. Moderate caffeinated coffee intake may therefore be a viable dietary strategy for managing constipation in the general population.

## Introduction

Coffee is one of the most popular and consumed beverages worldwide. The associations of coffee consumption with several diseases have been investigated. Coffee consumption has been linked with a reduced risk of several diseases, such as type 2 diabetes mellitus [1], cardiovascular diseases [2], liver outcomes [3–5], gallstone diseases [6], neurologic diseases [7–11], kidney function [12], prostate cancer [13], endometrial cancer [14], melanoma [15], non-melanoma skin cancer [16], colorectal cancer [17], as well as total mortality [18,19], suggesting its role in health promotion.

Chronic constipation is a highly prevalent global health issue that imposes a considerable economic burden and negatively affects the quality of life. The pooled prevalence of constipation is 14%, ranging from approximately 11% to 18% worldwide [20]. United States alone, constipation is responsible for about one million hospital visits each year [21]. Common risk factors for constipation include being female, advanced age, lower socioeconomic status, obesity, physical inactivity, diabetes, and low fluid intake [20,22,23].

The relationship between coffee consumption and constipation is poorly studied and controversial, with some studies suggesting a beneficial effect [24,25], while others reported no significant association [26]. These disparate results may be due to various factors, such as small sample size, unrepresentative population, inaccurate dietary measurements, different constipation definitions, and insufficient adjustment for confounders. In this study, we aim to examine the association of coffee consumption with constipation in a large representative sample of the U.S. population from the National Health and Nutrition Examination Survey (NHANES). Additionally, we also seek to explore the potential interactions of coffee consumption with other factors on constipation.

## Methods

### Study population

The National Health and Nutrition Examination Survey (NHANES) is a series of cross-sectional surveys conducted in the United States by the National Center for Health Statistics (NCHS). It employs a complex, multistage, clustered design to collect nationally representative data on the health and nutrition of the U.S. civilian, noninstitutionalized population. The NCHS Research Ethics Review Board approved the survey (Approval Number: #2005–06) and all of the participants provided written informed consent. All procedures were conducted in accordance with the relevant guidelines and regulations.

**Fig 1:**
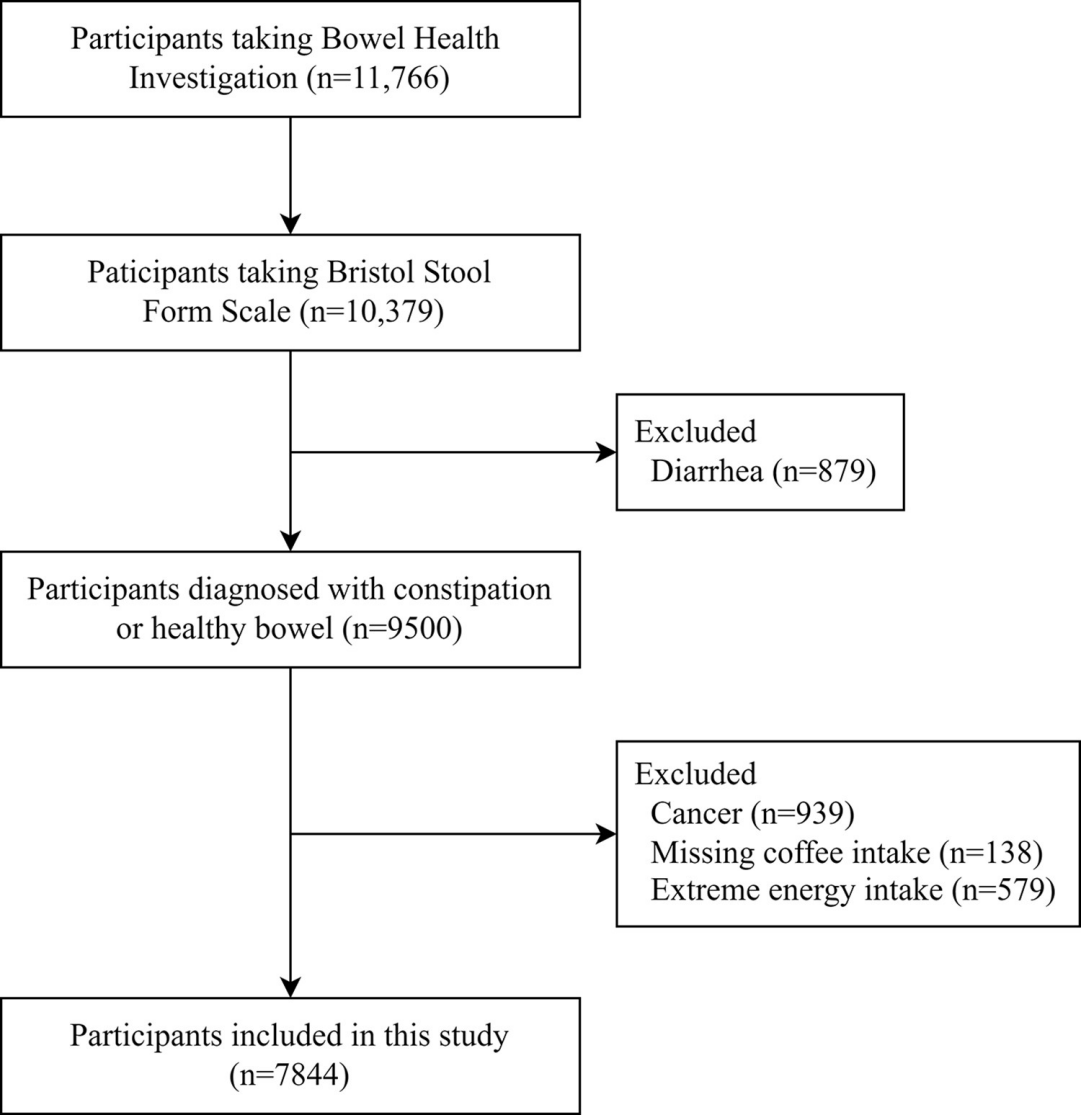
Flow chart of the study population. This figure depicts the study population screening process and the inclusion and exclusion criteria.

For the current study, the NHANES 2007–2008 and 2009–2010 cycles were combined. A total of 11766 participants aged 20 years or older who received an investigation for fecal incontinence and defecating function in a mobile examination center were identified. Among them, 10379 participants completed the Bristol Stool Form Scale (BSFS) questionnaire. After excluding participants diagnosed with diarrhea (n = 879), having a history of cancer (n = 939), with missing data on coffee consumption (138) and with extreme energy intake (daily energy intake <600 or > 3500 kcal for women and < 800 or >4200 kcal for men; n = 578), a total of 7844 participants were included in this study (Fig 1).

### Constipation definition

Constipation was assessed using the Bristol Stool Form Scale (BSFS), which is a valid and reliable measure for assessing stool form and has been used in many epidemiological studies [23,27–29]. The questionnaires were completed using a Computer-Assisted Personal Interview System in a Mobile Examination Center. Participants were asked to do the following: “Please look at this card and tell me the number that corresponds with your usual or most common stool type.” A card with colored pictures and descriptions of the seven BSFS types (Type 1–Type 7) was provided simultaneously for reference. Subjects who identified their usual or most common stool type as Type 1 (separate hard lumps, such as nuts) or Type 2 (sausage-like, but lumpy) were classified as having constipation. Those who reported their usual or most common stool type as Type 3 (like a sausage but with cracks in the surface), Type 4 (like a sausage or snake, smooth and soft) or Type 5 (soft blobs with clear-cut edges) were classified as having normal stool consistency.

### Assessment of coffee consumption

Coffee consumption was assessed through a single 24-hour dietary recall interview conducted in person at the Mobile Examination Center by trained dietary interviewers from the NHANES program. Coffee, caffeinated coffee, and decaffeinated coffee intake were transformed into standardized cup measurements, where one cup was equivalent to 6 fluid ounces. These intake levels were then categorized as Nondrinkers or <1 cup, 1–2 cups (≥1 cup and < 2 cups), 2–3 cups (≥ 2 cups and < 3 cups) and ≥3 cups.

### Covariates

We obtained self-reported data on age, sex, education, ethnicity, alcohol consumption, smoking, and vigorous physical activity. Alcohol drinking information was collected from questions about participants’ alcohol drinking behavior in their lifetime and past 12 months. Participants were classified into three groups: never (had < 12 drinks in lifetime), former (did not drink last year but drank ≥12 drinks in lifetime, or drank ≥12 drinks in one year and did not drink last year) and current (at least one drink last year). Smoking status was categorized into never, former, and current smokers. Vigorous physical activity was identified based on responses to two questions from the physical activity questionnaire. Participants were asked: (1) "Does your work involve vigorous-intensity activity that causes large increases in breathing or heart rate, such as carrying or lifting heavy loads, digging, or construction work for at least 10 minutes continuously?" and (2) "Do you do any vigorous-intensity sports, fitness, or recreational activities that cause large increases in breathing or heart rate, such as running or basketball, for at least 10 minutes continuously?" Participants who answered "Yes" to either of these questions were classified as engaging in vigorous physical activity. Body mass index (BMI) was calculated as the weight (in kilograms) divided by the square of height (in meters). Hypertension was determined through self-reported diagnosis, medication use, and/or an average systolic blood pressure of at least 130 mmHg or diastolic blood pressure of at least 80 mmHg. Diabetes was identified by self-report, insulin or medication use, and/or the level of glycohemoglobin HbA1c ≥ 6.5%. Depression was assessed using the PHQ9 questionnaire, with a score of 10 or more indicating depression [30].

As for dietary covariates, Healthy Eating Index 2015 (HEI-2015) scores were derived from the 24-hour dietary recall interview [31]. The HEI-2015 is a comprehensive measure of diet quality that assesses conformance to the Dietary Guidelines for Americans. It includes 13 components that cover various aspects of diet, such as total fruits, whole fruits, total vegetables, greens and beans, whole grains, dairy, total protein foods, seafood and plant proteins, fatty acids, refined grains, sodium, added sugars, and saturated fats. Higher HEI-2015 scores indicate a diet that aligns closely with dietary recommendations. By including HEI-2015 in our analysis, we account for a broad range of dietary factors, simplifying the model and reducing the risk of multicollinearity that could arise from including numerous individual dietary variables. Energy intake was also derived from the 24-hour dietary recall interview.

### Statistical analysis

Continuous variables were presented as mean (SE) and compared using the Wilcoxon rank-sum test; categorical variables were presented as percentages with SE and compared using the chi-squared test. To assess the association between coffee consumption and constipation, we applied two different approaches to capture both linear and nonlinear relationships.

Firstly, we employed restricted cubic splines (RCS) to assess the dose-response association between coffee consumption and the risk of constipation. In this analysis, coffee consumption was treated as a continuous variable, measured in cups per day.

Secondly, for logistic regression analyses, we categorized coffee consumption into four groups: nondrinkers or <1 cup, 1–2 cups, 2–3 cups, and ≥3 cups per day. This categorization was based on prior results from the RCS analyses. We generated three weighted logistic regression models to evaluate the odds ratio (OR) and 95% confidence intervals (95% CI) for the association between categorized coffee intake and constipation risk: (1) the initial model estimated the crude OR (Model 1); (2) Model 2 adjusted for year cycles, age, sex, ethnicity, education, and BMI; and (3) Model 3 further adjusted for alcohol drinking, smoking, vigorous physical activity, hypertension, diabetes, depression, HEI-2015 score, and energy intake. Subgroup analyses were also performed based on different levels of alcohol drinking due to the significant interaction observed with coffee intake.

All statistical analyses were performed using R version 4.3.0 and accounted for the complex survey design. P values < .05 were considered statistically significant.

## Results

### Participant characteristics

Table 1 shows the distribution of the participants’ baseline characteristics by total coffee consumption. The proportions of participants consuming no or less than 1, 1 to 2, 2 to 3, and ≥ 3 cups per day of total coffee were 54.5%, 16.1%, 10.3% and 19.1% respectively. For caffeinated coffee consumption specifically, the proportions were 57.5%, 15.1%, 9.6% and 17.9%, showing a higher proportion of participants consuming no or less than 1 cup per day. When it came to decaffeinated coffee, the majority of participants were non-drinkers or consumed less than 1 cup per day (96.6% for non-drinkers or <1 cup, 1.4% for 1–2 cups, 0.9% for 2–3 cups, 1.0% for ≥ 3 cups).

**Table 1:** Survey-weighted characteristics of the study population.

Characteristic	Nondrinkers or <1 cup (n = 4334)	1–2 cups (n = 1374)	2–3 cups (n = 846)	≥3 cups (n = 1290)	p-value
Age, mean (SE)	41.6 (0.42)	48.4 (0.59)	50.4 (0.81)	50.1 (0.58)	<0.001
Sex, % (SE)					<0.001
Female	53.3 (0.01)	58.4 (0.02)	49.4 (0.02)	44.8 (0.02)	
Male	46.7 (0.01)	41.6 (0.02)	50.6 (0.02)	55.2 (0.02)	
Race/ethnicity, % (SE)					<0.001
Non-Hispanic White	63.0 (0.03)	64.7 (0.03)	75.7 (0.02)	87.0 (0.02)	
Non-Hispanic Black	15.4 (0.02)	8.8 (0.01)	7.5 (0.01)	3.0 (0.01)	
Mexican American	9.8 (0.01)	11.2 (0.02)	7.9 (0.01)	3.9 (0.01)	
Other Hispanic	5.5 (0.01)	7.3 (0.01)	4.7 (0.01)	3.1 (0.01)	
Other Race	6.2 (0.01)	8.0 (0.01)	4.3 (0.01)	3.0 (0.01)	
Education, % (SE)					0.22
Less than high school	17.8 (0.01)	20.4 (0.02)	18.0 (0.02)	15.7 (0.02)	
High school	25.1 (0.01)	22.4 (0.02)	23.2 (0.03)	24.5 (0.02)	
Some college	32.2 (0.01)	29.4 (0.02)	29.1 (0.02)	30.7 (0.02)	
College or above	24.9 (0.01)	27.8 (0.02)	29.7 (0.03)	29.0 (0.02)	
BMI, Mean (SE)	28.8 (0.18)	28.1 (0.23)	29.1 (0.33)	28.3 (0.17)	0.03
Alcohol drinking, % (SE)					<0.001
Never	13.1 (0.01)	10.1 (0.01)	10.6 (0.02)	4.6 (0.01)	
Former	13.9 (0.01)	15.9 (0.02)	15.7 (0.02)	16.8 (0.02)	
Current	73.0 (0.01)	74.0 (0.02)	73.7 (0.02)	78.6 (0.02)	
Smoking, % (SE)					<0.001
Never	63.6 (0.01)	55.3 (0.02)	50.6 (0.02)	34.8 (0.02)	
Former	17.2 (0.01)	27.3 (0.02)	30.1 (0.02)	32.3 (0.02)	
Current	19.2 (0.01)	17.4 (0.01)	19.4 (0.02)	32.9 (0.02)	
Vigorous activity, % (SE)	41.0 (0.01)	35.1 (0.02)	37.3 (0.02)	41.4 (0.02)	0.04
Hypertension, % (SE)	43.7 (0.01)	46.4 (0.02)	52.4 (0.03)	49.7 (0.02)	0.009
Diabetes, % (SE)	8.9 (0.00)	12.3 (0.01)	11.6 (0.01)	9.5 (0.01)	0.02
Depression, % (SE)	7.7 (0.01)	5.8 (0.01)	7.1 (0.01)	8.7 (0.01)	0.14
HEI Index, mean (SE)	50.3 (0.45)	53.0 (0.49)	52.6 (0.50)	51.2 (0.79)	<0.001
Total energy (kcal), mean (SE)	2,064.4 (18.25)	1,933.4 (32.94)	2,043.9 (34.30)	2,107.9 (26.02)	0.001
Constipation	9.6 (0.00)	6.1 (0.01)	6.2 (0.01)	6.7 (0.01)	0.014

BMI: Body mass index; HEI Index: Healthy eating index.

Constipation prevalence varies across different levels of coffee consumption (p = .014), being highest among nondrinkers or those consuming less than 1 cup per day (9.6%), and lowest among those consuming 1–2 cups per day (6.1%). The average age of participants increased with higher levels of total coffee consumption (p < .001). Compared with women, men were more likely to be high coffee drinkers. A higher percentage of the Non-Hispanic White was observed as total coffee intake increased (p < .001). The BMI showed a slight variation across total coffee consumption groups (p = .03). As coffee intake increased, the percentage of individuals who never drank alcohol decreased, while the percentage of current drinkers increased (p < .001). There was a higher percentage of current smokers among those with higher total coffee consumption (p < .001). Among coffee consumers, the percentages of individuals engaging in vigorous physical activity and energy intake increased as the total coffee intake increased, while the HEI-2015 score decreased (p<0.05). Hypertension and diabetes also showed a slight variation across total coffee consumption groups (p<0.05).

### Dose-response relationship of coffee consumption with constipation

**Fig 2:**
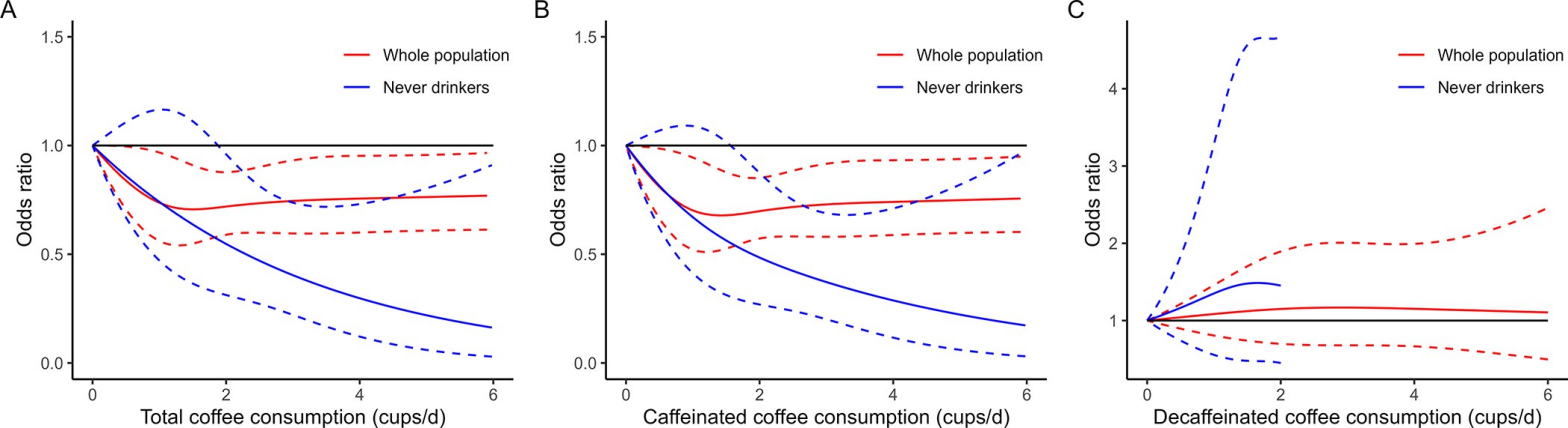
Restricted cubic splines for the association of coffee consumption with constipation in the whole population and among never drinkers of alcohol. A: Total coffee consumption and risk of constipation; B: Caffeinated coffee consumption and risk of constipation; C, Decaffeinated coffee consumption and risk of constipation. Models were adjusted for year cycles, age, sex, ethnicity, education, BMI, alcohol drinking (only for whole population analysis), smoking, vigorous activity, hypertension, diabetes, depression, HEI-2015 score and energy intake.

Fig 2 showed the dose-response relationships between the consumption of total coffee, caffeinated coffee and decaffeinated coffee with constipation adjusting for all covariates. The J-shaped association was observed for both total coffee and caffeinated coffee consumption with constipation (p for nonlinearity = 0.049 for total coffee and 0.0084 for caffeinated coffee), indicating an initial reduction in the risk of constipation at low to moderate levels of consumption, followed by a plateau or increase in risk at higher levels of consumption. The threshold for the lowest odds of constipation was approximately 1.5 cups/day for total coffee and 1.4 cups/day for caffeinated coffee. No significant relationship was observed between decaffeinated coffee consumption and constipation.

### Association of coffee consumption with constipation

To qualify the magnitude of relationships between coffee intake with constipation, we categorized coffee consumption (Nondrinkers or <1, 1–2, 2–3, and ≥3 cups/day) according to prior results of restricted cubic spline analyses. After controlling for potential confounders in Model 2, 1–2 cups/day of total coffee or caffeinated coffee was significantly associated with reduced risk of constipation (total coffee: OR 0.58, 95% CI 0.36–0.92, p = 0.02; caffeinated coffee: OR 0.55, 95% CI 0.36–0.84, p = 0.008) (Table 2). Upon further consideration of confounders in Mode 3, 1–2 cups/day of total coffee or caffeinated coffee was still associated with a lower risk of constipation although it is marginally significant for total coffee (total coffee: OR 0.61, 95% CI 0.35–1.06, p = 0.07); caffeinated coffee: OR 0.57, 95% CI 0.35–0.95, p = 0.03). No association was observed between decaffeinated coffee and constipation.

**Table 2:** Association of coffee consumption with constipation.

Characteristics	Mode 1		Model 2		Model 3	
	OR (95% CI)	p-value	OR (95% CI)	p-value	OR (95% CI)	p-value
Total coffee						
Nondrinkers or <1 cup	—		—		—	
1–2 cups	0.61 (0.40–0.93)	0.02	0.58 (0.36–0.92)	0.02	0.61 (0.35–1.06)	0.07
2–3 cups	0.62 (0.37–1.06)	0.08	0.70 (0.39–1.26)	0.21	0.73 (0.38–1.39)	0.29
≥3 cups	0.67 (0.51–0.89)	0.008	0.81 (0.60–1.10)	0.17	0.82 (0.57–1.18)	0.25
Caffeinated coffee						
Nondrinkers or <1 cup	—		—		—	
1–2 cups	0.57 (0.38–0.85)	0.007	0.55 (0.36–0.84)	0.008	0.57 (0.35–0.95)	0.03
2–3 cups	0.55 (0.34–0.90)	0.02	0.63 (0.36–1.09)	0.09	0.65 (0.36–1.19)	0.14
≥3 cups	0.69 (0.53–0.89)	0.007	0.84 (0.64–1.10)	0.18	0.85 (0.61–1.18)	0.28
Decaffeinated coffee						
Nondrinkers or <1 cup	—		—		—	
1–2 cups	1.34 (0.61–2.93)	0.46	1.15 (0.50–2.66)	0.72	1.22 (0.48–3.12)	0.64
2–3 cups	1.42 (0.50–4.04)	0.5	1.45 (0.48–4.37)	0.49	1.50 (0.42–5.37)	0.48
≥3 cups	0.62 (0.22–1.76)	0.35	0.75 (0.26–2.21)	0.59	0.70 (0.22–2.24)	0.51

Model 1: Unadjusted; Model 2: Adjusted for survey cycles, age, sex, ethnicity, education and BMI; Model 3: Additionally adjusted for alcohol drinking, smoking, vigorous physical activity, hypertension, diabetes, depression, HEI Index and total energy intake based on Model 2.

OR: Odds Ratio, CI: Confidence Interval.

### Subgroup analyses

We also detected the interactions between total coffee consumption and covariates and found the association of total coffee intake with constipation differed by alcohol drinking (p for multiplicative = 0.002, p for additive < 0.001). For participants who never drank alcohol, at least 3 cups/day of total coffee or caffeinated coffee showed a strong protective effect and was associated with an 88% risk reduction of constipation (Table 3). In addition, the associations of total coffee and caffeinated coffee with constipation shifted to inverse linear when restricted to never drinkers of alcohol (p for nonlinearity = 0.24 for total coffee, 0.13 for caffeinated coffee) (Fig 2). For participants who were current or former alcohol drinkers, no obvious association was observed.

**Table 3:** Association of coffee consumption with constipation by alcohol drinking.

Characteristic	Never		Current		Former	
	OR (95% CI)	p-value	OR (95% CI)	p-value	OR (95% CI)	p-value
Total coffee						
Nondrinkers or <1 cup	—		—		—	
1–2 cups	0.59 (0.31–1.14)	0.10	0.57 (0.28–1.18)	0.11	0.90 (0.50–1.63)	0.70
2–3 cups	0.79 (0.29–2.15)	0.62	0.66 (0.32–1.33)	0.21	1.12 (0.45–2.76)	0.79
≥3 cups	0.12 (0.02–0.68)	0.02	0.75 (0.43–1.30)	0.27	1.67 (0.85–3.29)	0.12
Caffeinated coffee						
Nondrinkers or <1 cup	—		—		—	
1–2 cups	0.54 (0.26–1.12)	0.09	0.53 (0.27–1.05)	0.07	0.90 (0.49–1.67)	0.71
2–3 cups	0.68 (0.23–2.07)	0.46	0.62 (0.32–1.22)	0.15	0.85 (0.32–2.23)	0.71
≥3 cups	0.12 (0.02–0.70)	0.02	0.80 (0.45–1.42)	0.41	1.51 (0.75–3.05)	0.22
Decaffeinated coffee						
Nondrinkers or <1 cup	—		—		—	
1–2 cups	1.00 (0.21–4.63)	>0.99	1.68 (0.49–5.76)	0.37	0.83 (0.17–3.92)	0.79
2–3 cups	2.55 (0.57–11.4)	0.19	1.14 (0.24–5.42)	0.85	2.22 (0.50–9.80)	0.26
≥3 cups	—	—	0.29 (0.06–1.45)	0.12	1.94 (0.48–7.77)	0.31

The associations were adjusted for survey cycles, age, sex, ethnicity, education and BMI, smoking, vigorous physical activity, hypertension, diabetes, depression, HEI Index and total energy intake.

OR: Odds Ratio, CI: Confidence Interval.

## Discussion

In this large, population-based cross-sectional study utilizing the NHANES data, we observed a J-shaped association between coffee consumption and risk of constipation in the whole population, with 1–2 cups/day of coffee being associated with lower constipation risk and high coffee consumption not being associated with constipation risk. Notably, the association became linear and inverse after restricting it to never drinkers of alcohol, indicating that the nonlinear relationship observed in the overall population was attributed to the interaction between coffee consumption and drinking. Furthermore, the protective effect of coffee was mainly driven by caffeine, as the association of caffeinated coffee with constipation was stronger either in the whole population or in the never drinkers of alcohol, while decaffeinated coffee showed no discernable relationship with constipation.

Murakami et al. [24] studied the association of coffee intake with self-reported constipation, finding a linear relationship among Japanese women aged 18–20 years. In contrast, Dukas et al. [25] reported a nonlinear relationship between coffee consumption and constipation defined by stool frequency in women, with six or more cups/day of coffee having a slightly increased risk for constipation, while one cup/day or less was inversely associated with constipation. Our results complement these studies and found an inverse but J-shaped relationship of coffee consumption with constipation defined by BSFS in the overall population aged 20 years old and this relationship became linear when restricting it to the never drinkers of alcohol.

Our findings underscore the importance of caffeinated coffee. While both caffeinated and decaffeinated coffee contain a range of bioactive compounds, only caffeinated coffee was associated with a lower risk of constipation. This result is in line with the known biological properties of caffeine, which can stimulate muscle contractions in the gastrointestinal tract, thereby enhancing bowel movements [32,33]. Caffeine promotes bowel movements by increasing the production of hormones like gastrin and cholecystokinin, which trigger the gastrocolic reflex, enhancing peristalsis and colon contraction [34]. It also releases catecholamines such as adrenaline and noradrenaline, further promoting bowel activity [35]. The lack of association between decaffeinated coffee consumption and constipation may thus reflect the absence of this key stimulant. It is worth noting that different brewing methods, types of coffee beans, roast level and preparation methods can affect the caffeine concentration. Therefore, the association between caffeinated coffee and constipation may vary and further studies should be considered.

Our study also unveiled that the relationship between coffee consumption and constipation varied with respect to alcohol drinking status. In participants who never drink alcohol, a linear and inverse relationship between coffee consumption and constipation was observed, while an association was absent among current or former drinkers. This may be explained by the fact that alcohol consumption can interfere with the absorption and metabolism of caffeine, thereby mitigating its effects [36]. Alcohol can also impair gastric emptying and intestinal motility, which may counteract the effect of coffee on constipation.

This study has several strengths. Firstly, our research drew upon nationally large representative data from NHANES, ensuring the results reflect a comprehensive view of the U.S. population. Secondly, we rigorously controlled a wide range of potential confounders, ensuring a more precise estimate of the relationship between coffee consumption and constipation. Thirdly, separate analyses were conducted for caffeinated and decaffeinated coffee, proving that the protective effect of coffee on constipation is attributed to caffeine. Lastly, a modification effect of drinking was observed in our study, indicating that the nonlinear relationship observed in the overall population was attributed to the interaction between coffee consumption and drinking.

Several limitations also need to be considered. Firstly, there is the possibility of reverse causation in our study. Individuals who consume more caffeinated coffee might also engage in other less healthy lifestyle habits, such as higher alcohol consumption and smoking, which could independently affect constipation risk. Conversely, individuals who opt for decaffeinated coffee might do so due to existing health conditions and might generally adhere to healthier lifestyle habits, potentially influencing their constipation risk. Although we controlled for various confounding factors in our analysis, including alcohol consumption, smoking, and physical activity, residual confounding cannot be entirely ruled out. Future longitudinal studies are warranted to further investigate the causal relationship between coffee consumption and constipation, considering the potential reverse causation and the role of underlying health conditions and lifestyle factors. Secondly, the assessment of coffee intake was based on a 24-hour dietary recall interview and thus recall bias is inevitable. Thirdly, the relatively small number of participants who consumed decaffeinated coffee compared to those who consumed caffeinated coffee might have influenced the observed lack of association between decaffeinated coffee and constipation. Fourthly, several additional confounders, such as average daily fluid intake, soft drinks, hot cocoa, energy drinks, and chocolate bars were not accounted for in this analysis. Future research should consider incorporating those factors to provide a more comprehensive understanding of dietary influences on constipation risk. Lastly, our results regarding the cutoff points of coffee consumption should be validated in other populations due to the potential for different distributions of coffee intake.

## Conclusion

In conclusion, consuming 1–2 cups of caffeinated coffee daily was associated with a reduced risk of constipation in the general population. Among never drinkers of alcohol, a linear protective effect was observed, with a notable 88% reduction in constipation risk for those consuming at least 3 cups per day. Moderate caffeinated coffee intake may therefore be a viable dietary strategy for managing constipation in the general population.

## Abbreviations

BMI: Body Mass Index
BSFS: Bristol Stool Form Scale
CI: Confidence Interval
HEI: Healthy Eating Index
NCHS: National Center for Health Statistics
NHANES: National Health and Nutrition Examination Survey
SE: Standard error
